# Interference effects of radical markings and stroke order animations on Chinese character learning among L2 learners

**DOI:** 10.3389/fpsyg.2022.783613

**Published:** 2022-08-11

**Authors:** Fengyun Hou, Xin Jiang

**Affiliations:** School of Psychology, Beijing Language and Culture University, Beijing, China

**Keywords:** radical, stroke order, Chinese character learning, L2 Chinese processing, second language acquisition

## Abstract

There is controversy around whether presenting sub-character units such as radicals and strokes are beneficial to L2 Chinese learning. The present study explored the effects of radical markings (i.e., marked radicals with different colors) and stroke order animations on learning Chinese characters. Forty Chinese L2 learners with native alphabetic languages were divided into high-and low-level groups. They were first required to learn Chinese characters under four conditions either: (a) presented radical markings with stroke animations; (b) presented no radical markings with stroke animations; (c) presented radical markings without stroke animations; or (d) presented neither radical markings nor stroke animations. After learning, the participants were given character recognition and character-meaning matching tests. Results showed that the presentation of radical markings increased the participants’ reaction times in the character recognition test and decreased their recognition accuracy. Moreover, presenting stroke order animations also decreased the participants’ accuracy in recognizing characters. Beyond that, presenting radical markings and stroke order animations had no significant influence on character-meaning matching tests. These results indicate that providing radical and stroke information might interfere with character learning instead of facilitating character learning. The results suggest that excessive visual information introduced in the learning process may increase L2 learners’ cognition load. Also, the findings contribute to theoretical arguments about the analytic and holistic processing of Chinese characters and the pedagogical implications for teaching Chinese as a second language.

## Introduction

A crucial aspect of vocabulary acquisition in a foreign language is to establish the interconnection among the three lexical constituents of orthography, phonology and semantics to develop a high-quality lexical representation (Perfetti and Hart, 2001, 2002; Perfetti et al., 2005). However, it is not easy to build up the lexical representations of a new language because of the differences in languages and their writing systems. For instance, Chinese characters present a stark contrast to alphabetic systems and provide distinctive challenges to Chinese second language learners (L2 learners) from alphabetic language backgrounds (e.g., Everson, 1998). Differing from alphabetical scripts, Chinese characters are composed of strokes (e.g., “一” and “|” are the first two strokes of character “苛”) and radicals (e.g., “苛” is composed of the radical “艹” on the top and the radical “可” on the bottom) in a complex two-dimensional configuration. Strokes are incrementally combined in a specific order, and different radicals are combined according to certain constructions. The complex internal structure of Chinese characters not only makes it more difficult for L2 learners to learn Chinese characters but also raises the interesting question as to whether providing information on strokes and radicals in the instructional process will contribute to the recognition of Chinese characters by L2 learners. In addition to the visual complexity, the correspondence between Chinese orthography, phonology and semantics is also different from alphabetic languages. Chinese has no grapheme-phoneme-correspondence and has numerous homophones, which means a given syllable can map to many different characters and has a lot of meanings (e.g., the syllable/dian4/ means both electricity and store). This implies that the orthography-semantic connection is more important and reliable than the orthography-phonology connection (Perfetti et al., 2005). Therefore, it is crucial for L2 learners to establish a robust orthography-semantic connection to be skilled readers.

Although strokes and radicals are two functional sub-character units involved in the character processing of native Chinese speakers (e.g., Peng and Wang, 1997; Li et al., 2005), it has been controversial as to whether the knowledge of strokes and radicals is beneficial for L2 learners to develop orthographic representations of Chinese character and connections between orthography and semantics. This question has been discussed from the perspective of Chinese character processing, exploring whether L2 learners process the strokes and radicals of Chinese characters in character recognition. In other words, these studies explore whether L2 learners process characters analytically or holistically. Some studies have suggested that L2 learners recognize Chinese characters using a holistic processing strategy that utilizes the overall information of the characters without analytically processing the strokes and radicals of the characters (Liu, 1993, 2008; An and Shan, 2007; Xie, 2015). Liu (2008) investigated the influence of stroke number and type of radical configuration on the recognition of characters by L2 learners. She found that neither the differences in the number of strokes nor the differences in the ways of combining radicals affected learners’ performance in character recognition. In other words, learners are more likely to adopt a holistic processing approach during reading. Acquiring the holistic form of characters is sufficient for learners to recognize characters and perform reading tasks, while decomposing characters into strokes and radicals increases the complexity of recognizing characters and reading (Liu, 2008; Xie, 2015). In contrast, some studies have argued that strokes and radicals, as two basic functional units, are involved in character processing among L2 learners (Taft and Chung, 1999; You, 2003; Feng et al., 2005; Shen, 2005; Hao, 2007; Shen and Ke, 2007; Jiang et al., 2020). Examining the stroke-number effect (e.g., Jiang et al., 2020) and radical position effect (e.g., Feng et al., 2005; Hao, 2007) in character processing by L2 learners, these studies have provided evidence to support that L2 learners employ the information from strokes and radicals for bottom-up and analytical processing. Therefore, introducing knowledge of strokes and radicals may facilitate learning Chinese characters and contribute to L2 character recognition.

In addition, a few studies have considered whether the character recognition levels and proficiency influence the character processing strategies that L2 learners adopt (Ke, 1998; Xu, 2007, 2009).^1^ Some studies concluded that as learners become more proficient in reading Chinese, the contribution of characters’ holistic form becomes greater (Xie, 2015), while others argued that it is the ability to decompose characters that develop as learners’ proficiency improves (See Footnote 1; Xu, 2007, 2009). Xu (2007) divided the development of decomposition ability of L2 learners into three different stages: (1) Stage zero, whereby learners are not capable of decomposing the internal structure; (2) Stage of perceptual decomposition, whereby learners have preliminary knowledge of the spatial structure of characters and have the basic ability to decompose characters; (3) Stage of structural decomposition, whereby learners are able to decompose the internal structure of characters proficiently. This three-stage developmental process implies that the development of character representations for learners may go through a process from holistic to analytical processing. This is inconsistent with findings of studies in children, which generally assume that children process characters from an analytical approach to a holistic one as their reading skills develop (e.g., Su and Samuels, 2010). As there is limited experimental research on character processing and development of the character representation, it is still difficult to determine whether L2 learners process characters holistically and whether L2 character processing strategies are related to the character recognition levels or not. These are the questions addressed in the present study.

Another issue that cannot be neglected in second language instruction is the cognitive load on learners. From the cognitive load perspective, it is vital to be aware of the potential cognitive overload and distraction resulting from providing information about strokes and radicals during instruction. According to cognitive load theory (Sweller, 1993), learning effectiveness can be affected when new information exceeds the learner’s limited working memory capacity (Baddeley, 1992). In other words, working memory may be overloaded when learners are required to process excessive amounts of information from instructional materials at the same time (e.g., Kalyuga et al., 1999; Chung, 2007). In addition, the simultaneous availability of multiple kinds of information may also cause the split attention effect (Chandler and Sweller, 1991, 1992, Owens and Sweller, 2008), further increasing the working memory burden. When processing multiple elements of information at the same time, learners need to spread their attention to focus on every element. It is shown that the split attention effect often arises when the holistic information has multiple elements which are difficult to understand independently (e.g., Sweller et al., 1998; Chung, 2007). These kinds of elements are required to be integrated mentally. Although one of the characteristics of Chinese characters is that they can be decomposed and recombined (Liu, 1993), focusing only on the internal components does not allow for a proper understanding of the holistic characters. This implies that when multiple levels of information, such as strokes and radicals, are emphasized simultaneously during instruction, L2 learners may not only be required to spread their attention on each level of elements but also to mentally integrate the information to reach comprehension. The process of decomposing and recombining may increase the complexity of the learning task and the difficulty of the corresponding mental operations (Liu, 1993). In this case, the provision of stroke and radical information may become an external cognitive load, which is not beneficial to L2 Chinese character learning.

Chinese characters can be decomposed into different levels of strokes, radicals and holistic characters, each containing various information such as the number of strokes, stroke order, radical position, radical function, etc. This study focuses on information about the stroke order and the radicals of Chinese characters in the up–down/right–left configuration.

The stroke order refers to the sequence in which individual strokes appear when writing Chinese characters. The stroke order effect has been found in the studies of Chinese character recognition with native speakers (e.g., Huang, 1986; Flores and Arcais, 1994; Qiu and Zhou, 2010; Yu et al., 2011). Stroke order is a part of the mental lexicon of characters for Chinese native speakers (Flores and Arcais, 1994). However, the stroke order of characters is significantly more sophisticated than the alphabet letters, making learning the stroke order a demanding task for L2 learners. Moreover, it is controversial about the necessity of teaching stroke and stroke order to L2 learners (e.g., Cao et al., 2013a). In the pedagogy of Chinese character teaching, a common practice for teaching stroke order is writing, and the positive influence of writing on both L1 and L2 reading has been supported by existing research (e.g., Tan et al., 2005; Guan et al., 2011; Cao et al., 2013a, 2013b; Xu et al., 2020). However, writing is time-consuming and labor-intensive (Allen, 2008). Stroke order animation as an auxiliary teaching tool, therefore, has become an economical alternative (Jin, 2003, 2006; Zhu and Hong, 2005; Chang et al., 2015). Nonetheless, research findings on the effectiveness of presenting stroke order animation for learning characters remain insufficient and inconsistent (Lu et al., 1999; Zhu and Hong, 2005; Jin, 2006; Zhu et al., 2012; Hsiung et al., 2017). Zhu and Hong (2005) examined whether the stroke order animations in multimedia flashcards are beneficial to learning Chinese characters and found that stroke order animations interfered with Chinese character learning. They inferred that L2 learners were overwhelmed by the excessive visual input in the stroke order animations, which distracted their attention and interfered with the memorizing of Chinese characters.

In contrast, a few studies have suggested that stroke order animations facilitate developing the orthographic representations and connections among orthography, phonology and semantics (Ng and Wu, 1990; Xu et al., 2013; Chang et al., 2015). To examine this hypothesis, Xu et al. (2013) compared the effectiveness of writing, stroke order animation, and passive character reading. They found that the three different approaches in the learning phase contributed to different aspects of character learning. The results from the lexical decision task show that writing and stroke order animations are more effective than passive reading in facilitating character recognition. In addition, as demonstrated by the meaning-matching task, reading improves meaning recall, and stroke order animations are more beneficial than writing in remembering the meaning of characters. Xu et al. (2013) explained the results in terms of the trade-off effect of the different learning approaches. Writing and stroke order animation guided learners’ attention to the low-level visual features of the characters. Yet both approaches reduce the attentional resources that learners invest in orthography-semantics association at the same time. They also pointed out that the trade-off effect is mitigated to some extent by the stroke animation, which enhances the learners’ visual orthographic representation as effectively as writing and supports the orthography-semantics connection better than writing.

Chang et al. (2015) also demonstrated that stroke order animation contributes to the development of learners’ orthographic representations and the trade-off effect between the three lexical components of Chinese characters. In this experiment, Chang et al. (2015) recorded the behavioral and ERP responses of the participants while they were learning and performing the old/new judgment tasks (i.e., character recognition test) and form-meaning matching tasks. They required the participants to learn the characters in either a dynamic (i.e., stroke order animations where the character is presented stroke by stroke) or a static (i.e., the whole character is presented at once) condition. Their behavioral results showed that participants required longer reaction times to recognize characters learned in the static condition than stroke order animations. In other words, the static presentation was more beneficial for developing the orthographic representation of the characters. In addition, there were no significant differences between the two conditions in the form-meaning matching task. However, the results of ERPs told a different story. The dynamic stroke order animation induced a larger P300 than the static presentation in the learning phase, indicating that the presentation of stroke order animations more effectively drew the learners’ attention to the incremental changes of the characters’ form during the learning process. On the contrary, the effect of P300 was not found in the character recognition test. Moreover, the N400 effect was found only in the static condition in the form-meaning task, indicating that the learners established a better connection between the orthography and semantics in the static condition than in the dynamic stroke order animation. This further demonstrated that learners allocated their attentional resources to orthography and semantics differently in different learning conditions. However, in this study, there was a difference in the exposures of characters between the two learning conditions. Because the complete form of characters was missing while presenting the stroke order animation, the processing time for the whole characters differed in the two conditions. This may lead to a disadvantage for the stroke order animation condition as shown by the behavioral results in the character recognition test. Furthermore, it may also explain why the P300 effect of the stroke order animations was only found in the learning phase but not in the post-tests. In sum, the role of stroke order animation remains unclear in developing orthographic representations and strengthening the connection between orthography and semantics.

Another sub-character unit of Chinese characters is the radical. As with strokes, the argument that radicals are a functional unit involved in Chinese character processing is well supported by the evidence from native speakers (e.g., Feldman and Siok, 1997; Peng and Wang, 1997; Taft et al., 1999; Zhang and Sheng, 1999; Zhou and Zeng, 2003). Although, as mentioned above, some of the studies have tended to support the hypothesis that L2 learners process characters holistically a few studies have argued that radicals are the functional units in character processing by L2 Chinese learners, from the perspective of both the character processing (e.g., You, 2003; Feng et al., 2005; Hao, 2007) and the character learning (Shu and Anderson, 1997; Taft and Chung, 1999; Chang et al., 2014; Xu et al., 2014). Research in support of the facilitation of radicals for L2 character learning suggested that radicals have relatively integrated structural features as opposed to many and varied strokes. As a result, the radicals can integrate the information at the stroke level (Taft and Chung, 1999) as well as direct learners’ attention to the internal structure of the character to some extent (Cao et al., 2013a). For instance, Taft and Chung (1999) required four groups of participants to learn Chinese characters in four different radical presentation conditions to investigate whether presenting radical information is beneficial for learning characters. They found that providing information about radicals facilitated the performance of learning characters’ form and meaning. They also found that it was most effective to provide the learners with information about the radicals when the characters were first presented, as opposed to presenting radicals systematically before learning characters or presenting radicals after learning characters.

Likewise, Xu et al. (2014) employed a classroom-based design and between-subjects to examine the effect of presenting radicals on Chinese character learning. Furthermore, they took learners’ language proficiency into consideration. In this experiment, each half of the participants at the beginning and intermediate levels were assigned to learn in the radical-based grouping condition, namely, the characters in each learning set shared the same radical (e.g., “婚” wedding, “嫁” to marry, “媳” daughter-in-law, “娃” baby, are sharing the radical “女” which means “female”), and the remainder were learned in the distributed condition, in which the characters within the same learning set had different radicals. They found that learning in the radical-based grouping condition for beginning learners significantly improved their performance in meaning recall and their radical generalized awareness in the radical recognition test compared to learning in the distributed condition. Characters sharing the same radical were also semantically related, which explains the better meaning recall by the beginning learners. However, there was no significant difference between the two conditions for intermediate learners. From this, Xu et al. (2014) inferred that the intermediate-level learners may have already developed a metalinguistic awareness regarding the internal structure of Chinese characters, enabling them to automatically decompose characters into sub-character units such as radicals without the instruction of explicit radical markings. This interpretation is in line with Xu (2007) assumption that L2 learners’ character processing undergoes a developmental process from holistic to analytical. Similarly, manipulating the comparison between the radical-based groupings and distributed conditions, Chang et al. (2014) further included four learning approaches namely handwriting, visual chunking (unlike splitting characters into radicals, the characters are decomposed into multiple chunks, e.g., “烟” smoke is divided into three chunks of “火,” “口,” “大”), passive-reading and stroke-reporting to explore effective ways of supporting orthographic learning at the beginning stages. Their result showed the advantage of visual chunking over other learning approaches in the radical-based grouping, indicating that presenting radical information is helpful in drawing learners’ attention to the decomposed sub-character units and supporting their orthographic learning (Cao et al., 2013a; Chang et al., 2014).

Previous studies either focused on when to provide L2 learners with the radical information (Taft and Chung, 1999) or focus on whether the same radicals need to be summarized for learners or not (Chang et al., 2014; Xu et al., 2014). However, to our knowledge, no existing research has explored whether marking radicals with different colors, a common practice in teaching scenarios, contributes to L2 orthographic and semantic learning. Moreover, experimental evidence on the role of radical information on Chinese character learning is still scarce, and most of the relevant studies employed the between-subject design (e.g., Taft and Chung, 1999; Cao et al., 2013a; Xu et al., 2014), which may involve confounding variables from the individual learners. In addition, no study has included both the stroke order animations and radical markings, the two daily teaching practices, in the investigation.

In the present study, we simultaneously examine the influence of the stroke order animations and radical markings on Chinese character learning. When providing radical information, we separate the compound characters into two radicals of left and right or top and bottom and then mark the different radicals in red and blue, respectively (e.g., the character “苛” is divided into the radical “艹” on the top which is marked in red, and the radical “可” on the bottom which is marked in blue). When providing stroke order animations, we used a similar methodology to Chang et al. (2015). However, in order to balance the issues caused by different exposure levels to the complete character between the conditions with and without stroke animations in their study, we keep the whole character as a light grey background during stroke order animation presentation. In other words, the stroke order animation refers to presenting the character stroke by stroke against a light grey character background. A 2 (with or without radical markings) × 2 (with or without stroke order animations) × 2 (the character recognition levels: high or low) mixed experimental design is employed in the present study. We recruit L2 learners with alphabetic language backgrounds and divide them into high-and low-level groups. The learners are required to learn characters in the four different presentation modes, and then take the immediate and one-week delayed post-tests. There are two tasks in the post-tests. One is the character recognition test which examines the orthography learning outcomes. Another is the character-meaning matching test which evaluates the effect of associating the orthography and semantics. The present study not only provides new experimental evidence regarding the influence of sub-character units such as radicals and strokes on the L2 character acquisition and L2 character processing, but also offers inspiration for teaching and learning characters as a second language.

## Materials and methods

### Participants

Based on previous studies (Xu et al., 2013; Chang et al., 2014), forty L2 Chinese learners participated in the experiment. According to an informal interview, all participants met the following criteria: (1) having an alphabetic native language, (2) being either a native English speaker or a nearly native English speaker who has learned English and used it for more than eight years, (3) not coming from a family of Chinese heritage, (4) not having a learning or reading disability, (5) being right-handed, (6) possessing a normal or corrected-to-normal vision. All the participants are undergraduate and postgraduate students from Beijing Language and Culture University, Beijing Normal University and Beijing Foreign Studies University. They were learning and using Chinese daily when taking part in experiments. All participants were given informed consent before undertaking the experiment and reimbursed for their time. Ethical approval for the experiment was obtained from the Institutional Review Board of Beijing Language and Culture University.

The participants were divided into two groups based on their character recognition levels. Character recognition level was defined as the amounts of known characters of the L2 learners, which were measured by recognizing the given characters and writing down the corresponding Pinyin. To measure learners’ levels of Chinese character recognition, all the participants were given a character recognition test of 100 characters before the experiment. These characters for the character recognition test were selected from the Balanced Corpus of Modern Chinese from the State Language Commission of China.^2^ We sorted all the characters of Corpus by their frequency from highest to lowest. Then the 100 characters were randomly selected from the first 3,000 characters and sorted according to their character frequency. Participants were asked to write down as many of the given characters in Pinyin as possible. The scoring is in line with previous research (Jiang, 2003). The accuracy of the tone of the characters was not scored. For example, 1 point would be scored for writing down “shang” for the character “上.” They were then assigned to either high-or low-level groups according to the character recognition test results, with one half in the high-level group and the other half in the low-level group. Statistics show that there is significant difference on character recognition test scores [*t* (38) = −0.811, *p* < 0.001] between the high-level group (score range: 51 ~ 86, *Mean* = 62.1, *SD* = 10.72) and low-level group (score range: 13 ~ 49, *M* = 34.85, *SD* = 10.53). Apart from this, there were no significant differences between groups regarding genders (7 males and 13 females in each of the two groups) or ages (high-level group: 23 ~ 30, *Mean* = 23, *SD* = 3; low-level group: 23 ~ 29, *Mean* = 23, *SD* = 3.03; *t* (38) = 0.11, *p* = 0.91 > 0.10).

### Materials

One hundred and twenty low-frequency (Character frequency: *Mean* = 2.81/million, *SD* = 3.25) Chinese characters were selected and translated into English as learning materials. The characters were selected from the Balanced Corpus of Modern Chinese from the State Language Commission of China.^3^ Half of the target characters had a left–right structural configuration (e.g., 淳) and the other half had an up-down configuration (e.g., 苛). To ensure the participants did not know the target characters before the learning phase, ten L2 learners were asked to rate the familiarity of the 120 characters on a 5-point scale, with “1” referring to very unfamiliar and “5” to very familiar, from 1 to 5, indicating a gradually increasing familiarity with the character. All these learners were from alphabetic language backgrounds and have high character recognition test scores (*M* = 72.7, *SD* = 12.1). The rating result showed that the familiarity of all target characters is 2 or below (*M* = 1.4, *SD* = 0.31).

**Figure 1:**
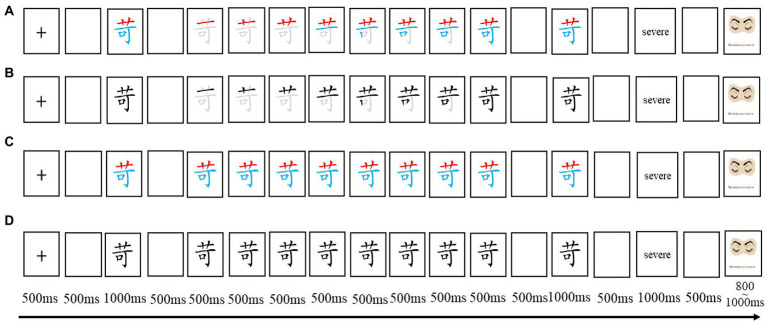
Provides an example of the four different presentation modes of the same character and the learning procedure. The four different presentation conditions are: **(A)** presented radical markings with stroke animations; **(B)** presented no radical markings with stroke animations; **(C)** presented radical markings without stroke animations; or **(D)** neither presented radical markings nor stroke animations.

The key manipulation of the learning materials is the four different presentation modes of target Chinese characters. We used Microsoft Word and PowerPoint to create radical markings and stroke animations. The radical markings refer to the two radicals of the characters displayed in red and blue, respectively, while stroke animations indicate that the characters are presented stroke by stroke in the writing order against a light grey character background. In the learning phase, the characters were presented in four different kinds of presentation modes, either: (a) presented radical markings with stroke animations; (b) presented no radical markings with stroke animations; (c) presented radical markings without stroke animations; or (d) presented neither radical markings nor stroke animations. Figure 1 provides an example of the four different presentation conditions of the same character. All participants experienced all four learning conditions. A Latin square was used to counterbalance the learning conditions across participants.

The experiment consisted of a pre-test and two different kinds of post-tests, namely a character recognition and character-meaning matching test. The pre-test includes 40 high-frequency characters as fillers in addition to the 120 target learning characters to prevent participants from developing a response strategy. Apart from the 120 target characters that had already been presented in the learning phase, 120 low-frequency characters that had not appeared in the learning phrase were added to the character recognition post-test as distractors. The distractors were selected from the same Corpus as the target materials. We matched the character frequency (targets: *Mean* = 2.81/million, *SD* = 3.25; distractors: *Mean* = 2.8/million, *SD* = 3.38) and the stroke numbers (targets: range = 5 ~ 14, *Mean* = 9.57, *SD* = 2.22; distractors: range = 5 ~ 14, *Mean* = 9.23, *SD* = 2.12). Statistical results show that there is no significant difference in either character frequency [*t* (238) =0.04, *p* = 0.97] or the stroke numbers [*t* (238) =1.22, *p* = 0.23] between the targets and the distractors. In addition, identical to the target characters, half of the distractors have a left–right structural configuration, and the other half had an up-down configuration. The participants were asked to determine whether the character had been learned or not. In the character-meaning matching test, each character corresponds to a pair of English interpretations, one of which is the correct translation of the character and the other from the meaning of another character.

### Procedures

The entire experimental procedure consisted of a character recognition test, a pre-test, a learning phase, an immediate post-test and a one-week delayed post-test. The procedure for each phase is specified below.

#### Character recognition test

Participants were given a paper and pencil test in which they were asked to write down as many of the given characters in Pinyin as possible without being timed.

#### Pre-test

To further confirm that the participants did not recognize the target characters to be learned, we conducted a pre-test before the learning phase, whereby we asked the participants to read the recognized characters aloud. Characters that participants were able to read prior to learning would be excluded from the data analysis.

#### Learning phase

The participants were instructed to learn the characters by following the presentation modes on the screen and remembering the characters and their English translation. Each character learning trial proceeded as follows: a 500 ms fixation, a blank for 500 ms, a target character presented for 1,000 ms, another blank for 500 ms, and a presentation of the character according to different conditions (the presentation duration of characters is equivalent in the four conditions with each stroke presented for 500 ms in the stroke order animation condition), a blank for 500 ms, the target character presented again for 1,000 ms, another 500 ms blank, presentation of the character’s English translation for 1,000 ms followed by a final 500 ms blank. At the end of a trial, an eye image was displayed, and the participants were instructed to press the “space bar” to continue. Figure 1 provides the learning procedure for the same character under the 4 conditions. Each one of the four conditions was presented in a block. 30 characters randomly present in each condition block. Before the beginning of each block, there was a practice character to familiarize the participants with the presentation mode and the experimental procedure. The participants experienced all four conditions, and the sequence of learning conditions was counterbalanced. 120 characters are learned three times in three separate blocks, each containing four conditions. Learning blocks were separated by a short break.

#### Immediate and delayed post-test

**Figure 2:**
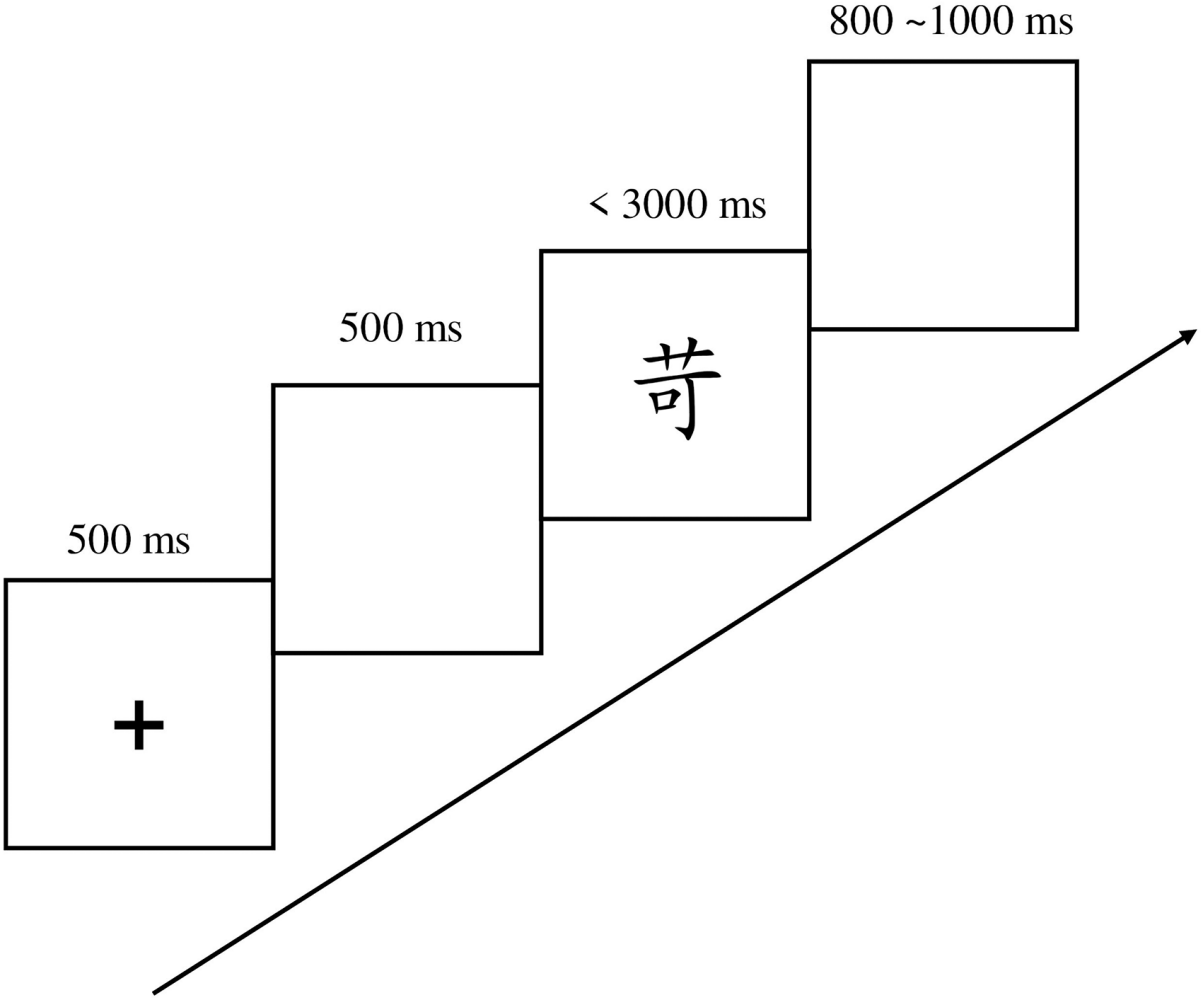
Provides the procedure for the Chinese recognition test. Participants were instructed to judge whether the character has been presented in the learning phase as quickly and accurately as possible.

**Figure 3:**
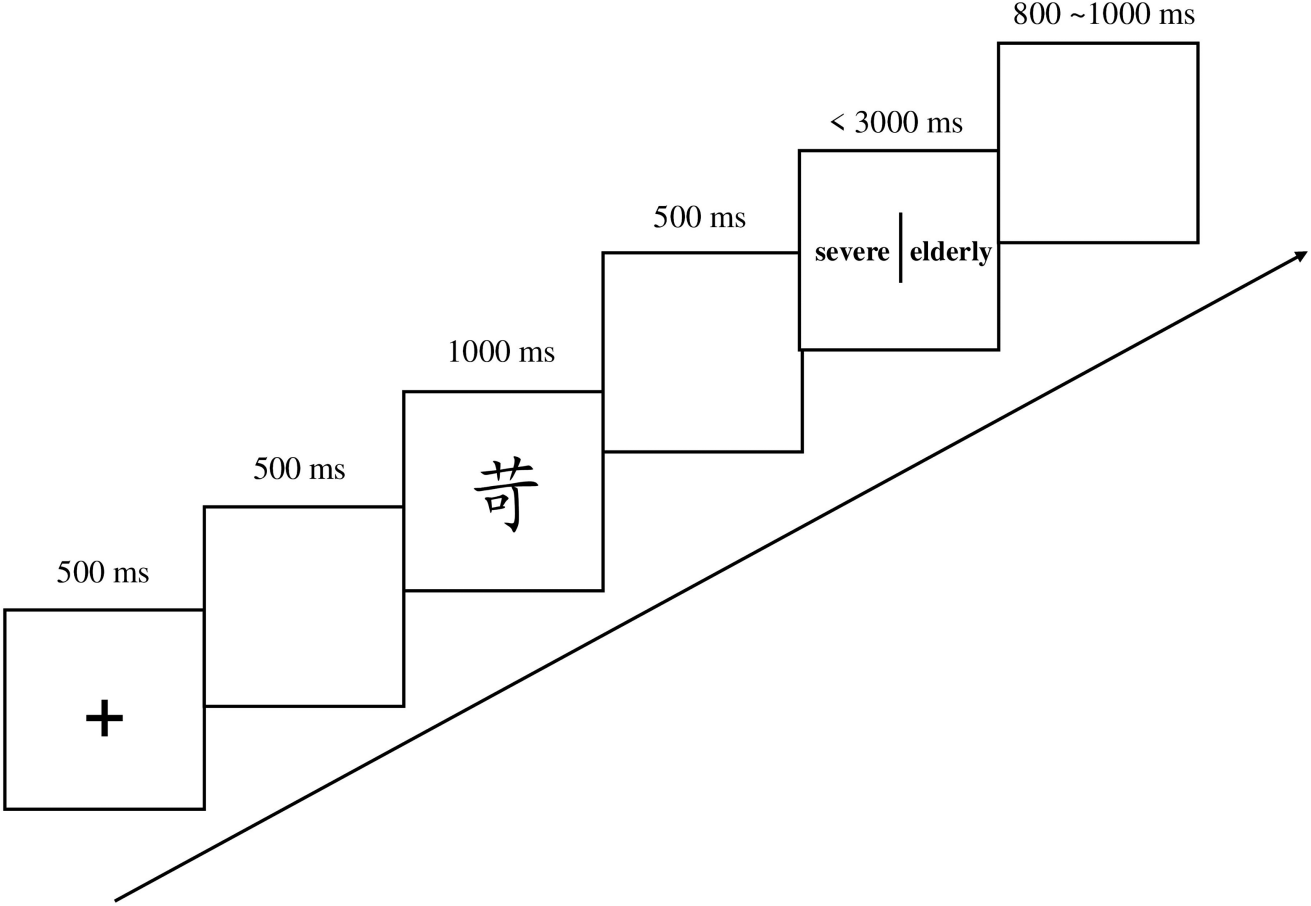
Provides the procedure for the character-meaning matching test. Participants were instructed to decide which English word was the correct translation of the presented character as quickly and accurately as possible.

The experimental procedures for the immediate and one-week delayed post-tests both contain a character recognition test (Figure 2) and a character-meaning matching test (Figure 3). In the character recognition test, a fixation was presented for 500 ms, and then a blank screen for 500 ms, a character was then presented on the center of the screen. The participants were instructed to judge whether the character had been presented in the learning phase as quickly and accurately as possible. In the character-meaning test, a fixation was presented for 500 ms followed by a 500 ms blank screen, then a character was presented for 1,000 ms. After another 500 ms blank screen, two English words was showed on the screen and are separated by a vertical line. The participants were then required to decide which English word was the correct translation of the presented character as quickly and accurately as possible.

All the computerized procedures were programmed and carried out on E-Prime software.

## Results

Tables 1 and 2, respectively, present the descriptive statistics of the accuracy and reaction times of the two groups of participants under the four different character presentation conditions in the immediate test. Tables 3 and 4 present the descriptive statistics in the delayed tests. For each participant, characters that the participant knew before attending the experiment were excluded from the data analysis based on the results of the pre-test (0.15%). The repeated-measures ANOVAs were conducted on the analysis of the reaction time and accuracy in both the immediate and delayed tests.

**Table 1:** Mean accuracy (%) and standard deviation (in parentheses) of the immediate post-test.

	Character recognition levels	With stroke order animations		Without stroke order animations	
		With radical markings	Without radical markings	With radical markings	Without radical markings
The character recognition test	Low-	82.00 (0.12)	86.17 (0.12)	85.00 (0.08)	86.17 (0.11)
	High-	85.31 (0.07)	88.32 (0.11)	88.16 (0.83)	89.98 (0.77)
Character-meaning matching test	Low-	84.83 (0.15)	83.67 (0.10)	85.67 (0.10)	85.67 (0.11)
	High-	87.14 (0.08)	86.66 (0.11)	86.64 (0.11)	89.83 (0.07)

**Table 2:** Mean reaction times (ms) and standard deviation (in parentheses) of the immediate post-test.

	Character recognition levels	With stroke order animations		Without stroke order animations	
		With radical markings	Without radical markings	With radical markings	Without radical markings
The character recognition test	Low-	1,031.23 (193.95)	1019.42 (200.98)	1044.67 (204.12)	1011.29 (181.12)
	High-	883.59 (146.29)	861.93 (133.46)	890.39 (141.65)	837.98 (124.08)
Character-meaning test matching test	Low-	862.60 (219.16)	866.33 (203.57)	888.37 (199.38)	888.03 (230.12)
	High-	773.00 (228.75)	765.46 (220.02)	764.79 (214.36)	761.13 (224.42)

**Table 3:** Mean accuracy (%) and standard deviation (in parentheses) of the one-week delayed post-test.

	Character recognition levels	With stroke order animations		Without stroke order animations	
		With radical markings	Without radical markings	With radical markings	Without radical markings
The character recognition test	Low-	59.33 (0.13)	62.33 (0.15)	63.50 (0.16)	62.33 (0.13)
	High-	66.08 (0.23)	68.61 (0.17)	66.63 (0.22)	68.59 (0.19)
Character-meaning matching test	Low-	73.67 (0.11)	75.00 (0.10)	73.33 (0.10)	73.17 (0.10)
	High-	75.70 (0.16)	76.67 (0.13)	77.95 (0.11)	73.97 (0.13)

**Table 4:** Mean reaction times (ms) and standard deviation (in parentheses) of the one-week delayed post-test.

	Character recognition levels	With stroke order animations		Without stroke order animations	
		With radical markings	Without radical markings	With radical markings	Without radical markings
The character recognition test	Low-	1132.71 (312.89)	1107.91 (313.76)	1097.32 (324.98)	1065.36 (194.85)
	High-	993.78 (255.49)	967.15 (240.60)	983.00 (264.54)	976.93 (276.15)
Character-meaning matching test	Low-	970.52 (229.70)	955.96 (216.31)	977.96 (219.81)	957.60 (211.70)
	High-	896.24 (290.09)	913.87 (295.15)	884.84 (282.81)	884.05 (267.29)

In the immediate test, trials with incorrect responses and trials with reaction times less or greater than 3 standard deviations were rejected from the analysis of reaction time (15.1% in the character recognition test and 14.27% in the character-meaning matching test). Results of the analysis of the reaction times showed a significant main effect of radical markings [*F* (1, 38) = 11.547, *p* = 0.002, *MSE* = 35563.153, *ηp^2^* = 0.233] in the character recognition test. The presentation of radical markings significantly increased reaction time for learners to recognize characters compared to the presentation of characters without radical markings. In addition, we found that character recognition levels have a significant main effect with the participants in the low-level group having longer recognition times than those in the high-level group [*F* (1, 38) = 9.677, *p* = 0.004, *MSE* = 1000798.961, *ηp^2^* = 0.203]. Apart from these, no other significant main effects or interactions were found in either the character recognition or character-matching tests on the analysis of reaction time (*ps* > 0.05).

ANOVAs on mean accuracy showed a significant main effect of stroke animation [*F* (1, 38) = 4.192, *p* = 0.048, *MSE* = 0.014, *ηp^2^* = 0.093] in the character recognition test. The presentation of stroke animation significantly decreased recognition accuracy for learners compared to the presentation of characters without stroke animation. Additionally, the significant main effects of radical markings [*F* (1, 38) = 12.507, *p* = 0.0001, *MSE* = 0.026, *ηp^2^* = 0.248] revealed that presenting the radical markings decreased the participants’ accuracy in the character recognition test, compared to not presenting the radical markings. Apart from these, the analysis of accuracy did not show other main effects or interactions in either the character recognition or character-meaning matching tests (*ps* > 0.05).

In the one-week delayed test, we applied the same criteria for data exclusion as in the immediate test. Trials with incorrect responses and reaction times less or greater than 3 standard deviations were rejected from the analysis of reaction times (35.73% in the character recognition test and 25.42% in the character-meaning matching test). ANOVAs performed on reaction times and accuracy showed no significant effect in either character recognition or character-meaning tests (*ps* > 0.05).^4^

## Discussion

The present study includes both radical markings and stroke order animations as within-subject variables to explore their effects on developing the character orthographic representations and the orthography-semantics connections for L2 learners at the different character recognition levels. In the immediate post-test, we found that participants in the high-level group had shorter reaction times than those in the low-level group in the character recognition test. In addition, the critical findings are, surprisingly, that participants had shorter reaction times and higher accuracy when recognizing characters that were learned without radical markings than those learned with radical markings. Moreover, the participants were more likely to correctly recognize the characters learned without stroke order animations than those learned with stroke order animations. These results suggest that providing learners with radical markings and stroke order animations fails to facilitate learning characters for L2 learners, but may instead interfere with their character learning, especially with respect to acquiring the orthography representations of characters. Apart from the above, we did not find significant differences resulting from the presence or absence of radical markings and stroke order animations in the character-meaning matching tests. Given that the stroke order animations and radical markings used in the present study are not only common practices in L2 Chinese instruction, but also the common ways of presenting characters in some multimedia self-learning software (e.g., Chen et al., 2013; Chang et al., 2015), their negative effect deserves more attention in both experimental studies and pedagogical discussion.

Consistent with our results, the interference effect of providing the information of stroke orders has been found in previous studies (e.g., Zhu and Hong, 2005; An and Shan, 2007; Hsiung et al., 2017). For instance, Hsiung et al. (2017) found that stroke order animations were insufficient to improve the effectiveness of character recognition or meaning learning for L2 learners. Unlike character processing by native speakers, the stroke order animations probably do not play a role in the L2 learners’ mental lexicon of characters. The existing studies have found the stroke order effect in native speakers’ character processing (e.g., Huang, 1986; Flores and Arcais, 1994; Qiu and Zhou, 2010). They suggested that this effect could be attributed to the fact that native speakers consistently write characters following the stroke order. Thus, the stroke order information becomes a kind of sensory-motor memory derived from writing (Longcamp et al., 2003; Cao et al., 2013a) and then stored in native speakers’ orthographic representations of characters. On the contrary, L2 learners lack the long-term experience of writing characters in stroke order. As a result, the stroke order cannot become a part of their mental representations after a short period of visual learning. In addition, the presentation of stroke order animations does not involve sensory-motor memory. It in turn cannot provide sensor-motor information to facilitate the development of their orthographic representations of characters. Therefore, presenting learners with stroke order animations may not be able to facilitate learning Chinese characters, but instead increases the complexity of the learning task.

Furthermore, another reason for the negative effects of the stroke order animations may arise from the overload of cognitive load and the splitting of attention. In the study conducted by Zhu and Hong (2005), stroke order animations in flashcards produced a split-attention effect on L2 learners’ character memorization. That is, because the available attentional resources in each sensory channel are limited, the interference effect of stroke order animations may be attributed to the redundant visual information introduced by the animations (Zhu and Hong, 2005; Zhu et al., 2012). Our experiment presented the stroke order animations whilst simultaneously displaying the complete characters as background. This design balanced the difference in the amount of the holistic character exposures between the conditions presented with and without stroke order animations (Chang et al., 2015). However, it may have also resulted in overwhelming participants with too much visual information and therefore distracted learners’ attention from learning the forms of characters.

As with the stroke order animation, the interference by excessive visual information may also appear in the radical markings. The negative effect of presenting radicals in our findings is inconsistent with some previous studies (e.g., Taft and Chung, 1999; Chang et al., 2014; Xu et al., 2014). A possible reason is that the method we used to present the radical information is different from previous studies. Instead of presenting radicals at the different stages of character learning (Taft and Chung, 1999) or presenting by radical-based grouping (Chang et al., 2014; Xu et al., 2014), we used different colors to mark radicals and presented them throughout the learning process. The colors are designed to direct learners’ visual attention to the internal structure of the characters. However, colors may have also produced a negative impact as an additional distracting factor. The complex visual information may have forced participants to add a process for matching radicals to the colors, causing the split attention effect. Consequently, it leads to a negative impact on learners’ character learning.

Both the stroke order animations and the radical markings required L2 learners to decompose and recombine Chinese characters. The negative results from the process of decomposition and combination are consistent with Liu (1993) study. Liu (1993) suggested that decomposing the radicals during L2 instruction increases the complexity of the learning task and the corresponding difficulty of the mental operations. In our experiments, stroke order animations and radical markers are presented to direct learners’ attention to decomposing characters. However, the characters ultimately need to be understood as a holistic unit by L2 learners. When multiple elements of a Chinese character are presented simultaneously during instruction, the splitting attention effect may be increased as these elements are difficult to understand independently and need to be integrated mentally to reach comprehension (Sweller et al., 1998). This process of decomposing and combining may lead to longer reaction times and lower accuracy in our results.

In addition, from the perspective of L2 learners’ encoding and processing strategies, the character recognition task may only require learners to acquire and apply the holistic information of characters (Liu, 2008; Xie, 2015). In our experiment, L2 learners’ best performance was achieved under the condition that neither stroke order animation nor radical marking was presented. This possibly can be explained by the encoding specificity principle (Tulving and Thomson, 1973). According to the encoding specificity principle (Tulving and Thomson, 1973), it would be easier to retrieve the learned information if the testing approach matched the encoding format. In our character recognition test, we presented the characters in the same way as in the condition without the stroke order animations and radical markings. Thus, the best memory performance was obtained by memorizing characters in the manner closest to the test approach, as it helped to retrieve information in the same context as the test approach.

Moreover, our results tend to support the theoretical argument that L2 learners adopt a strategy of holistic processing when recognizing Chinese characters rather than analyzing the strokes and radicals (e.g., Liu, 1993, 2008; An and Shan, 2007; Xie, 2015). Research regarding L2 character processing suggests that L2 learners adopt a holistic processing strategy to recognize characters, especially when performing the simple character recognition task (Liu, 2008). In our character recognition test, we asked participants to identify whether the characters have been learned in the experiment or not. Participants were likely to have been able to recognize characters based only on their familiarity with the holistic form of the characters. Previous studies have investigated learners’ strategies for character learning and found that the most common strategy used by learners was to memorize the holistic form of the characters and then make connections between the holistic character form and the existing schema in the brain to enhance memorization (Jiang and Zhao, 2001). This strategy can also be observed in the common writing errors of L2 learners (e.g.,Gao, 2001; Jiang and Liu, 2004). There is a large proportion of writing errors caused by missing strokes and incorrectly written radicals (Gao, 2001; Jiang and Liu, 2004). In this case, emphasizing the information of radicals and stroke orders may not be beneficial for learners, but instead may result in the interference effect on their orthographic learning of holistic characters that we found in the present study.

Some studies of L2 character processing have suggested that learners of different language proficiencies have different approaches for processing Chinese characters (e.g., See Footnote 1; Xu, 2007, 2009). According to the three-stage developmental theory proposed by Xu (2007), we should expect that the stroke order animations and radical markings would have different effects on the two groups of learners with different character recognition levels. Although we found a significant difference in reaction times between the high-and low-level groups regarding recognizing characters, we did not find any interaction among the stroke order animations, radical markings and character recognition levels. A possible reason is that after studying the characters three times, both the high-and low-level groups achieved a great learning effect for the target characters (in the character recognition test, the accuracy was higher than 80% under all presentation conditions). This question regarding the effect of character recognition levels on L2 character processing is worth exploring in more depth in future studies.

Another interesting question is the influences of stroke order animations and radical markings on semantic learning. That is, whether the manipulation of orthographic information affects the connections between orthography and semantics in L2 character learning. Previous studies have found that, for L2 learners, presenting the complete form of characters is better for learning the meaning of characters than emphasizing the information of sub-character units (Xu et al., 2013; Chang et al., 2015). Based on the lexical constituent model (Perfetti and Hart, 2002; Perfetti, 2007), they explained these results as a trade-off effect between the lexical constituents of orthography and semantics (Xu et al., 2013; Chang et al., 2015). In other words, the emphasis on orthographic information in the learning phase yields the advantage of orthographic learning, but reduces the cognitive resources for semantic learning simultaneously, thus affecting the establishment of linking orthography and semantics (Chang et al., 2015). However, according to the character-meaning matching test, we did not find any significant effect due to stroke order animations and radical markings on semantic learning. This may be attributed to our test approach. Chang et al. (2015) required participants to make yes-no judgments about the English meaning of the characters. In contrast to their testing approach, participants were asked to select the correct English meaning out of two in our character-meaning matching test. Also, both English meanings were taken from the learning target material. Thus, participants could have adopted various strategies, for example, the exclusion strategy, to make choices without necessarily correctly establishing form-meaning associations. This simple test approach may have to a certain extent prevented us from observing the effect of stroke order animations and radical markings on the character-meaning matching test. Future research may need to revise and improve the test to explore the effects of the stroke order animations and radical markings on learning character meanings.

The present study shows that learning characters’ orthography under the conditions of presenting stroke order animations and radical markings was less effective. In terms of pedagogical implications, does this mean we should abandon these approaches in L2 Chinese character instruction or place less emphasis on the internal structural information of Chinese characters? We consider the negative effects worthy of concern, but it may be reckless to abandon these teaching methods. Instruction in stroke order and radical differentiation may not facilitate rapid recognition of Chinese characters but may benefit other aspects of Chinese character learning, which were not apparent in our character-recognition task. For example, Tong and Yip (2015) have found that L2 learners encode orthographic, phonological, and semantic information of radicals during processing, and that the development of radical sensitivity and generalization skills contribute to the reading skills of L2 learners. A sensible solution is categorizing the characters required to be learned according to different teaching objectives, such as elementary recognition and mastery of orthography, phonology, and semantics. For characters categorized in the teaching objectives of elementary recognition, it may be better to introduce less information about the internal structure of the characters, such as stroke order and radical position. Instead, it may be beneficial to help L2 learners become familiar with these characters through multiple presentations of holistic characters.

Moreover, the negative effects of stroke order animations and radical markings in the present study may also be attributed to cognitive overload. This raises considerations about the external cognitive load of instructional design. Chinese character is an entirely different writing system for learners from alphabetic script backgrounds. L2 learners may inherently experience a considerable cognitive load in learning and processing the newly learned Chinese characters. Therefore, when designing Chinese character instructional materials or multimedia learning software, the cognitive load of L2 learners should be thoroughly considered. For instance, it is necessary to consider that visually presenting multiple information simultaneously may lead to split attention because of the limited cognitive resources in a single modality (Mayer and Moreno, 1998; Zhu and Hong, 2005; Zhu et al., 2012). How do we minimize the external cognitive load of instructional materials and learning software while at the same time effectively guiding learners to decompose and combine the internal structures of Chinese characters such as strokes and radicals? This is an important question that warrants exploration by both second language acquisition researchers and teachers.

In addition, there is another reason why it is too early to say that we should abandon these teaching practices. That is, the positive effects of stroke order animation and radicals may have occurred during the learning process but were not revealed in our post-test results. The learning of strokes and radicals could be a mediating process to improve the character recognition and the overall character recognition levels of L2 learners. In the present study, we measured the learning outcomes for orthography and semantics *via* two different tasks in post-tests. Although both stoke order animations and radical markings show negative effects in the character-recognition test, it is still possible that they play a role in supporting L2 learners to move from analytical to holistic processes and improve their character recognition levels. Our experiments could not provide evidence for this question because the behavioral measures such as reaction times and accuracy only provide information after the character processing is complete. The study conducted by Chang et al. (2015) have provided insightful inspiration. They used ERP to explore the role of stroke order animations in the character learning phase. Their behavioral results were consistent with the present study, showing that stroke order animations negatively affected character recognition. However, their ERP results showed that the condition with stroke order animations induced a greater P300 amplitude than the presentation without stroke order animations during the learning phase. P300 reflects the mental representation updating process driven by attention (Donchin, 1981), implying that L2 learners allocated more attentional resources to process characters under the guidance of stroke order animations during the learning phase. ERP has high temporal resolution and enables real-time recording during language processing (Chang et al., 2015). This technique is a powerful tool which can be utilized by researchers to explore the L2 character learning processes further. Moreover, it will contribute to the investigation of the underlying mechanisms of L2 character learning and processing and the mediating process of sub-character unit information in the learning process. In future studies, we will apply ERP to explore further the influences of stroke order animations and radical markings on L2 character learning.

## Conclusion

In this study, we used stroke order animations and radical markings for emphasizing orthographic information in sub-character units and explored their effects on L2 character learning. We found that the presentation of the stroke order animations and radical markings during the learning phase had a negative effect on L2 character recognition. These results may be due to the additional load of the visual information from the stroke order animations and radical markings. Additionally, these results may reflect the holistic processing strategy adopted by L2 learners. This study provides theoretical contributions for L2 character acquisition and pedagogical implications for L2 Chinese character instruction.

## Data availability statement

The raw data supporting the conclusions of this article will be made available by the authors, without undue reservation.

## Ethics statement

The studies involving human participants were reviewed and approved by the Institutional Review Board of Beijing Language and Culture University. The patients/participants provided their written informed consent to participate in this study.

## Author contributions

FH and XJ proposed the ideas and designed the study. FH performed the experiments, analyzed the data, and wrote the manuscript. XJ provided the comments and modified the manuscript. All authors contributed to the article and approved the submitted version.

## Funding

This study was funded by a grant from the National Social Science Fund of China (17ZDA305), a grant from the Project of School of Psychology at Beijing Language and Culture University (19YJ130004), which was from the Fundamental Research Funds for the Central Universities, and a grand from the Discipline Team Support Project of Beijing Language and Culture University (GF201906) to the second author.

## Conflict of interest

The authors declare that the research was conducted in the absence of any commercial or financial relationships that could be construed as a potential conflict of interest.

## Publisher’s note

All claims expressed in this article are solely those of the authors and do not necessarily represent those of their affiliated organizations, or those of the publisher, the editors and the reviewers. Any product that may be evaluated in this article, or claim that may be made by its manufacturer, is not guaranteed or endorsed by the publisher.

## Footnotes

^1^Ke, C. (1996). A model for Chinese Orthographic Awareness. Unpublished manuscript.

^2^ http://corpus.zhonghuayuwen.org/

^3^ http://corpus.zhonghuayuwen.org/

^4^We further included the testing time as a factor in the ANOVAs and analyzed the reaction time and mean accuracy of the Chinese character recognition test. The results of a repeated measures ANOVA on reaction times show that there are significant main effects of radical markings [*F* (1, 38) = 12.53, *p* = 0.001, *ηp*^2^ = 0.25] and testing time [*F* (1, 38) = 6.87, *p* = 0.013, *ηp*^2^ = 0.153]. These results are consistent with the previous results of running two models. The statistical results of mean accuracy revealed a significant main effect of radical markings [*F* (1, 38) = 9.45, *p* = 0.004, *ηp*^2^ = 0.20], a marginally significant main effect of stroke order animations [*F* (1, 38) = 3.27, *p* = 0.079, *ηp*^2^ = 0.08] and a significant main effect of testing time [*F* (1, 38) = 106.04, *p* < 0.001, *ηp*^2^ = 0.74]. In addition, there is no significant interaction between different character presentation modes and the testing time, which means that the influence of different presentations of Chinese characters on maintenance effects could not be reflected in the present study.
